# Suitability of Solvent-Assisted Extraction for Recovery of Lipophilic Phytochemicals in Sugarcane Straw and Bagasse

**DOI:** 10.3390/foods11172661

**Published:** 2022-09-01

**Authors:** Francisca S. Teixeira, Lígia L. Pimentel, Susana S. M. P. Vidigal, Paula T. Costa, Manuela E. Pintado, Luís M. Rodríguez-Alcalá

**Affiliations:** CBQF—Centro de Biotecnologia e Química Fina—Laboratório Associado, Escola Superior de Biotecnologia, Universidade Católica Portuguesa, Rua Diogo Botelho 1327, 4169-005 Porto, Portugal

**Keywords:** sugarcane, straw, bagasse, lipids, glycolipids, 1-octacosanol, phytosterols, ethanol

## Abstract

Sugarcane is primarily harvested to meet up to 80% of global sugar demand. Recently, lipids recovered from their biomass (straw and bagasse) have attracted much attention due to their possible utilisation in biofuel production but also by the presence of health-promoting compounds as phytosterols (i.e., improvement of cardiovascular function) or 1-octacosanol (i.e., anti-obesity). Although this fraction is commonly obtained through solid–liquid isolation, there is scarce information about how different solvents affect the composition of the extracts. This research work aimed to study whether, in sugarcane straw and bagasse samples, Soxtec extraction with widely used dichloromethane (DCM) would be suitable to recover most of the lipid classes when compared to other available solvents such as food grade ethanol (EtOH) or solvents without regulation restrictions for food and drug applications (i.e., acetone and ethyl acetate). The obtained results allow concluding that sugarcane waxes from straw and bagasse are complex lipid mixtures of polar and non-polar compounds. According to the extraction yield, the best results were obtained with ethanol (5.12 ± 0.30% and 1.97 ± 0.31%) for both straw and bagasse, respectively. The extractant greatly influenced the lipid composition of the obtained product. Thus, DCM enriched the isolates in glycerolipids (mono-, di- and triglycerides), free fatty acids, fatty alcohols, fatty aldehydes, phytosterols and hydrocarbons. On the other hand, EtOH resulted in polar isolates rich in glycolipids. Therefore, depending on the application and objectives of future research studies, the solvent to recover such lipids needs to be carefully selected.

## 1. Introduction

Humankind faces a situation in which an increasing population (8.5 billion by 2030 and 9.7 billion in 2050) will continue to increment the demand for both energy and natural resources [1]. Thus, the need to bring sustainability policies applied to social, economic and environmental aspects has resulted in the development of the concepts of circular, green and bioeconomy [2]. The latter focuses on the reutilisation of biowaste (i.e., biological resources from the land and sea) to produce food, energy and materials [3].

A promising source of biowastes are sugarcane plantations (*Saccharum officinarum* L.). Sugar cultivation has increased from 1721 million tonnes in 2008 to 1907 million tonnes in 2018, 731 million tonnes of which came from Brazil and 337 million tonnes from India [4]. This harvest is primarily oriented to meet 80% of total sugar demand [5] and during the period 2018/2019, production reached 179.3 million tonnes of sugar [6]. The rest of the crop mass yields residues from the agricultural (i.e., tops and straw) or industrial steps such as filter cake, molasses, and bagasse [7,8].

Sugarcane lipids have also recently attracted significant attention as different approaches have demonstrated their suitability in the framework of the bioeconomy. As it is a high biomass crop, it could theoretically outperform oil production when compared to traditional oilseed harvests (i.e., soya), therefore representing a means of decreasing biofuel production costs [9]. Moreover, sugarcane oil accumulates in the leaves and stems and it is composed of phospholipids (PLSs)—that can be eliminated with acetone during biofuel production—in addition to triglycerides (TGs) and free fatty acids (FFAs), the latter of which are converted into diglycerides (DGs) [10]. Moreover, other initiatives have demonstrated how genetic engineering can optimise lipid production to reach up to 20% [11]. This is indeed an important breakthrough as the regular content varies from 0.1% to 0.3% depending on the variety, although the composition is far more complex since it includes fatty alcohols (FOHs), phytosterols, ketosteroids, hydroxyketosteroids and terpenoids [12].

Such a profile may represent new opportunities as some of those compounds have been associated with beneficial effects for human health. Works recovering lipids from sugarcane leaves through supercritical fluid extraction (SFE) have shown yields of 1.6 g lipid/100 g leaves and sterol contents of 1.09 g/kg leaves [13]. According to EU regulations, plant sterols contribute to the maintenance of normal blood cholesterol levels when the daily intake is of at least 0.8 g [14]. Those investigations also reported the presence of other bioactive compounds such as policosanol (i.e., a mixture of long-chain FOHs) where octacosanol (FOH 28:0) was present in concentrations of 1.9 g/kg of rind, 0.7 g/kg of bagasse and 0.1 g/kg of leaves [13]. However, other authors reported that an FOH 28:0 content in hand-peeled rind from different sugarcane cultivars varied from 0.5 g/kg to 1 g/kg [15].

Indeed, FOH 28:0 has been suggested as a potential ingredient for the prevention and treatment of obesity and related metabolic disorders. Thus, mice treated with a diet rich in fat and supplemented with FOH 28:0 (doses of 60 mg/kg/day) resulted in lower body weight and fat gain, insulin resistance and hepatic lipid content by increasing brown tissue activity and improving the hepatic lipid metabolism [16]. Moreover, Lee et al. (2019) [17] conducted a study on the impact of FOH 18:0 supplementation in taekwondo athletes (40 mg/d during 6 days) subjected to rapid weight loss through caloric restriction with high-intensity exercise training. This study reported that there was an improvement in the lipid profile which was accompanied by an increase in the high-density lipoprotein (HDL) levels and a decrease in low density lipoprotein (LDL) and TGs. A reduction in oxidative stress (increase in superoxide dismutase) was also observed when compared to the control group. Additionally, in mice exposed to a cage change strategy to induce mild stress and sleep disturbance, doses of 100–200 mg FOH 28:0/kg clearly restored the stress-affected sleep [18].

In those research works studying the lipid fraction collected from sugarcane biomass, isolation was performed by either supercritical fluids [13] or by means of Soxhlet with hexane: methanol (20:1 *v*/*v*) [15], carbon tetrachloride [19] or acetone (AcO) [20]. Likewise, limonene can be used as a solvent to extract sugarcane wax thanks to its lower toxicity, flammability and environmental risk when compared to conventional solvents such as n-hexane [21].

In lipid research, Soxhlet extraction has been widely used for solid–liquid isolation, although it presents the drawback that the process usually extends over several hours. However, the Randall or immersive (in boiling solvent) method (also commercially called Soxtec ^TM^) was originally developed, and later recommended by the Association of Official Analytical Chemists (AOAC) to isolate fat from meat and meat products and drastically reduce the operation time (i.e., 30 min) [22]. However, further collaborative studies have shown that this method is also suitable to recover lipids from vegetable biomass [23].

The methods described by Folch et al. [24] and Bligh and Dyer [25] represent the gold standard in lipid isolation but these involve the use of toxic solvent mixtures (i.e., chloroform:methanol) and several steps (i.e., re-isolation, filtration, collecting of lipid-containing layer) and are therefore also time-consuming. On the other hand, they were originally intended for animal tissues on the basis that halogenated solvents would recover most of the lipids while methanol would result in protein precipitation. Regarding sugarcane lipids, some authors have assayed those procedures described by Hara and Radin [26] for analytical purposes in bagasse, stem and juice [27].

Although most of the available data describing the composition of sugarcane lipids were obtained by the analyses of samples after the solid–liquid extraction, the range of assayed solvents is generally quite limited. In the framework of lipid extraction from sugarcane biomass for a further assessment of their health-promoting properties, it is important to understand how solvents affect the composition of a lipophilic fraction [28]. Moreover, in order to move towards more sustainable processes, it is important to assess the impact of analytical or food-grade solvents and how these affect extracts’ bioactivity [29]. 

Thus, the authors of this research work hypothesised that different solvents such as dichloromethane are efficient means of recovering lipids such as FOHs, TGs or hydrocarbons, although these are also capable of isolating PLSs. Nevertheless, others such as acetone are not suitable for isolating polar lipids. Furthermore, it would be useful to test solvents that are less toxic than those originally proposed (i.e., dichloromethane) or those allowed by the EU food and drug regulations [30,31], which can be obtained in food grade or can be produced through sustainable processes such as ethanol (EtOH) or recently acetone (AcO) [32].

Accordingly, the aim of this research investigation was to study the effect of solid–liquid extraction using dichloromethane (DCM), EtOH, AcO or ethyl acetate (EtAc) on the lipid composition of sugarcane bagasse and straw. The obtained results show that the composition of the extracts is greatly influenced by the solvent, and therefore, the solvent must be carefully selected depending on its application. The reported result will allow future research studies to perform a greener and less time-consuming extraction method using a more efficient and selective solvent for the extraction of bioactive compounds.

## 2. Materials and Methods

### 2.1. Materials and Chemicals

Sugarcane straw and bagasse were provided by Raizen, (Brotas, Brazil) and collected in August 2019. The samples were dried at 50 °C in a ThermoFisher Scientific oven (Waltham, MA, USA) until they reached a constant weight, milled in a Retsch SM 100 Cutting Mill and sieved in a Retsch Sieve AS 200 (Haan, Germany) to a 315 < x < 900 µm size. 

Extraction solvents including EtOH (F.C.C. Food Grade 96% (*v*/*v*)) were purchased from PanReac AppliChem (Barcelona, Spain), AcO (ACS Grade ≥99.5%) from Merck (Darmstadt, Germany), EtAc (HPLC-Grade ≥99.7%) from Honeywell (Charlotte, NC, USA) and DCM (HPLC Grade ≥99.9%) from VWR Chemicals (Radnor, PA, USA). 

For HPLC assays, the mobile phases were prepared with the following solvents: 2-propanol (LC–MS Grade ≥99.9%) and isooctane (HPLC Grade ≥99.8%) purchased from VWR Chemicals (Radnor, PA, USA), AcO (HPLC-Grade ≥99.8%) from ThermoFisher Scientific (Waltham, MA, USA), EtAc (HPLC-Grade ≥99.7%), water (for HPLC) from Honeywell (Charlotte, NC, USA), acetic acid (HPLC Grade ≥99.8%) from Carlo Erba Reagents (Barcelona, Spain) and triethylamine (TEA) (≥99.5%) from Merck (Darmstadt, Germany).

The internal analytical standard tetracosane (99%) and the derivatising reagent N, O-Bis(trimethylsilyl) trifluoroacetamide with 1% trimethylchlorosilane (BSTFA), used for the GC-MS analysis, were purchased from Merck (Darmstadt, Germany).

### 2.2. Lipid Extraction 

The lipophilic extraction was carried out in triplicate using the Foss Soxtec ^TM^ 8000 apparatus (Hilleroed, Denmark). The dried straw and bagasse samples (3 g) were extracted with EtOH, DCM, AcO and EtAc at 1:20 (*w*/*v*). The extraction temperature was adjusted according to the solvent: 130 °C for EtOH and EtAc; 110 °C for AcO; and 90 °C for DCM. The extraction period comprised 2 h of boiling and 1 h of rinsing at atmospheric pressure. Afterwards, the solvents were evaporated at Heidolph HeiVAP value digital rotatory evaporator (Schwabach, Germany) under reduced pressure in a temperature-controlled bath at 40 °C. The obtained extracts were weighed and the yields assessed. 

### 2.3. High-Performance Liquid Chromatography–Evaporative Light Scattering (HPLC–ELSD) 

The samples were accurately weighed and dissolved in dichloromethane to a concentration of 5 mg/mL. Afterwards, samples were analysed on an HPLC (model 1260 Infinity II; Agilent Technologies, Santa Clara, CA, USA) attached to an evaporative light scattering detector (ELSD; 1290 Infinity II, Agilent Technologies, Santa Clara, CA, USA) using nitrogen as a nebulising gas coupled to a Zorbax RX-SIL column (Agilent; 2.1 × 150 mm, 5 µm). Analysis conditions were assayed as described by Abreu et al. [33], with some changes. Four mobile phases were used with the following compositions: A, isooctane:ethyl acetate (99.8:0.2, *v*/*v*); B, acetone:ethyl acetate (2:1, *v*/*v*) containing 0.1% acetic acid (*v*/*v*); C, 2-propanol:water (85:15, *v*/*v*) containing 0.013% acetic acid (*v*/*v*) and 0.031% of TEA *v*/*v*; and D, EtAc.

The flow rate was set at 0.275 mL/min with the gradient composition described in Table S1. and an injection volume of 20 µL. The detector was set as follows: the evaporator and nebuliser temperature was set to 60 °C with nitrogen as the nebulising gas at a 1.20 SLM flow rate.

### 2.4. Gas Chromatography –Quadrupole Mass Spectrometry (GC–QqQ) 

Samples were previously derivatised into their trimethylsilyl derivatives (TMSs). Thus, in a glass vial, 5 mg of lipid extract was accurately weighed and added to 100 uL tetracosane (0.5 mg/mL in DCM) and 30 µL of BSTFA. The mixture was incubated during 60 min at 30 °C and DCM was then added to a final volume of 1.3 mL. 

Derivatised samples were analysed on a GC–QqQ model EVOQ (Bruker, Karlsruhe, Germany) mass spectrometer, with a Rxi-5Sil MS column (30 m × 250 µm × 0.25 µm nominal) at constant flow of 1 mL/min. The carrier gas used was helium and the operating conditions were as described by Attard et al. [13] with some modifications. The injector was set to 330 °C, the oven temperature started at 60 °C with a hold for 5 min, then the temperature was increased at 3 °C/min until 330 °C and maintained for 20 min. The mass spectrometer detector was operated in electron ionization mode (EI) at −70 eV, the source temperature of 280 °C, the transfer line at 300 °C and a quadrupole in a scan range of 33–1000 amu per second. The compound identification was based on the comparison of the obtained mass spectra with the information on the NIST library (v. 2.3) as well as by comparison with the reference compounds.

### 2.5. Fourier Transform Infrared Spectroscopy with Attenuated Total Reflectance (FTIR-ATR) 

The samples were analysed on a PerkinElmer Paragon 1000 FTIR (Waltham, MA, USA) with the ATR accessory. The spectra were obtained in the wavenumber range of 4000–550 cm^−1^, with a resolution of 4 cm^−1^, by accumulating 16 scans.

### 2.6. Differential Scanning Calorimetry (DSC) 

**Figure 1:**
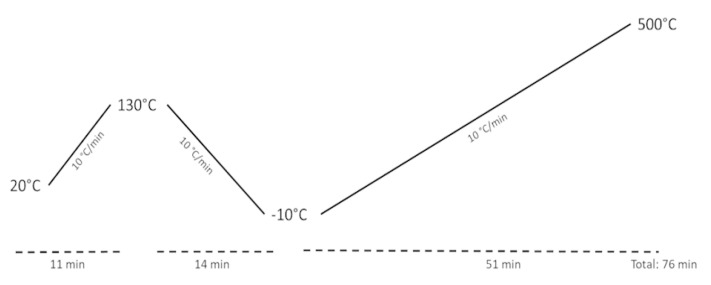
DSC temperature programme.

The thermal characteristics of the samples (melting, crystallisation, oxidation and decomposition temperatures) were measured on a 204 F1 Phoenix DSC (Netzsch, Germany). The samples were weighed (4 mg) in a pierced lid aluminum pan and analysed under an N_2_ flow of 40 mL/min, using the temperature program described below (Figure 1). First, the sample thermal history was eliminated by heating from 20 °C to 130 °C. Then, cooling to −10 °C followed by a second heating from −10 °C to 500 °C. The heating and the cooling were performed at a constant rate of 10 °C/min. Only the transitions observed during the cooling and the second heating cycles were taken into account. 

### 2.7. Statistics

Results are reported as mean values ± standard deviation. Data were first analysed for normality distribution. Levene’s test was applied to verify the homogeneity of the variances. Afterwards, a one-way ANOVA test was applied with a Tukey post hoc test to determine the differences within groups. The level of significance was set in general at 0.05. Analyses were performed with the aid of the Jamovi software (v 1.6.3.0; The Jamovi project (2020)), retrieved from https://www.jamovi.org, accessed on 13 January 2021).

## 3. Results and Discussion

### 3.1. Extraction Yields

Straw and bagasse from sugarcane were extracted with different solvents (ethanol, EtOH; acetone, AcO; ethyl acetate, EtAc; dichloromethane, DCM) by Soxtec ^TM^. As mentioned in the Introduction, previous studies have demonstrated that sugarcane produces healthy lipophilic phytochemicals [13,20]. However, lipid isolation is a complex and cumbersome task that involves the utilisation of organic solvents, usually biphasic systems, which are toxic [34] and complicates their utilisation as bioactives [29]. The current research work aimed to assess the recovery of lipids from sugarcane straw and bagasse using accelerated solid–liquid isolation (e.g., Soxtec^TM^) to reduce both the time and the possibility to alter the lipids and improve solvent recovery [35]. Moreover, halogenated solvents such as chloroform or DCM have been widely used in the isolation of lipids but their toxicity has raised much concern and currently, research is directed towards its replacement [36]. Thus, in this research investigation, solvents that are allowed by the EU food and drug regulations [30,31], that can be obtained in food grade or can be produced through sustainable processes such as EtOH or more recently AcO [32], were selected. 

**Figure 2:**
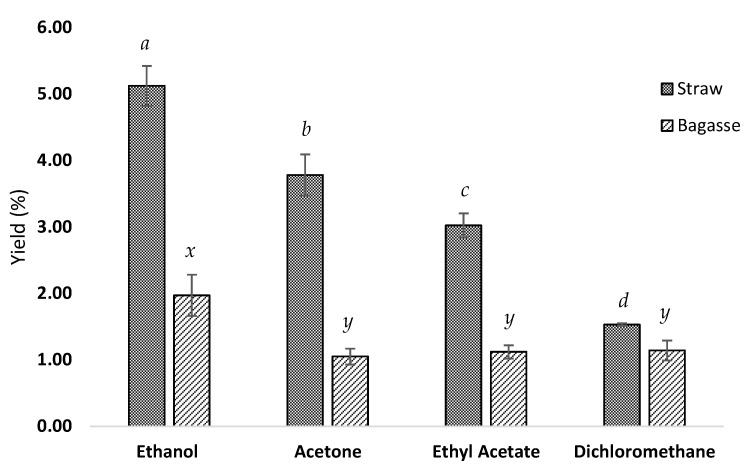
Lipophilic extraction yields (%) of straw and bagasse samples with different solvents. Different letters indicate statistically significant differences between solvents (*p* < 0.05). a, b, c and d for straw samples and x and y for bagasse samples.

After obtaining the extracts, the first approach to understand the suitability of the different tested solvents was to evaluate the isolation yields. These are presented in Figure 2. According to those results, the highest yields were obtained using ethanol: 5.12 ± 0.30% and 1.97 ± 0.31% for straw and bagasse, respectively. Straw extraction with acetone achieved a higher yield than ethyl acetate: 3.78 ± 0.31% (AcO) and 3.02 ± 0.18% (EtAc), respectively, while the dichloromethane extraction showed the lowest yields (1.53% and 1.14%, respectively, for straw and bagasse). Therefore, for the straw samples, all the results were significantly different (EtOH > AcO > EtAc > DMC, *p* < 0.05), while for bagasse, EtOH resulted in the best recovery (*p* < 0.05) while AcO > EtAc and DCM showed no significant differences. This clearly points towards a matrix effect affecting AcO, EtAc and DCM. 

Results on the utilisation of supercritical fluids to recover wax from rind, leaves and bagasse reported elsewhere resulted in yields of 1.60% for leaves, followed by the rind (0.8%) and the lowest for bagasse (0.53%) [13]. Other authors performed the isolation by Soxhlet for 16 h using AcO and recovered 1.4% in straw samples and 0.9% in bagasse [20]. However, other studies found no differences in the lipid content between the straw and bagasse (0.66% vs. 0.62%, respectively) when using isopropanol/hexane mixtures [27]. This highlights the relevance of the condition selected for the extraction of lipids. 

Such differences in extraction yields using different solvents are clearly associated with their polarity since lipids have a wide scope of different chemical properties. In fact, DCM was the extraction solvent with the lowest polarity and also the one that obtained the lowest yield, probably as it is more suitable to extract non-polar compounds. On the other hand, other solvents despite their higher polarity such as EtOH exerted the best performance since these can extract both polar and non-polar lipids [37], covering a greater range of molecules and therefore resulting in higher extraction yields. 

Accordingly, to deepen our knowledge of how solvents affect the lipid distribution in the obtained extracts, their analysis by HPLC–ELSD was then assayed. 

### 3.2. Lipid Classes Profile of the Assayed Samples by HPLC–ELSD

To the best of our knowledge, few previous studies have reported the lipid profile of sugarcane by-products. Thus, in research works conducted on hand-peeled sugarcane rinds and cane stalk samples, hexane–methanol Soxhlet extracts were composed of fatty aldehydes (FALs), sterol esters, TGs, FOHs, FFAs and sterols (ST) [15]. According to those results, FALs and sterol esters were resolved in the same peak and were the main compounds. On the other hand, further investigations showed that the lipid fraction of sugarcane stems was composed of TGs, FFAs, plant stanols and STs, glycolipids (i.e., monogalactosyldiacylglycerol and sterol glycosides) and phospholipids (PLSs—phosphatidylethanolamine, PE; phosphatidylcholine, PC; and phosphatidylinositol, PI) [27].

In the current research work, different extracts obtained after assaying EtOH, AcO, EtAc and DCM in the straw samples were analysed by HPLC–ELSD (Table 1). The results show that the main lipid moiety for AcO, EtAc and DCM samples was that corresponding to the elution of hydrocarbons. The highest concentration was detected in DCM (37.24 ± 1.07 g/100 g lipids) and AcO (31.95 ± 3.62 g/100 g lipids) followed by EtAc (29.96 ± 1.35 g/100 g lipids), while the lowest amount was found in EtOH (24.36 ± 0.06 g/100 g) (*p* < 0.05). In this latter sample (i.e., EtOH), the glycolipids content was 34.98 ± 1.66 g/100 g lipids, while it was 23.63 ± 2.18 g/100 g lipid and 23.25 ± 1.00 g/100 lipids for the AcO and EtAc samples (*p* < 0.05), respectively. Otherwise, the DCM extracts showed the significantly lowest content (10.55 ± 1.83 g/100 g).

In bagasse samples (Table 2), extracts followed the same trend as compounds in the hydrocarbon chromatographic region were prominent in DCM samples (38.93 ± 4.93 g/100 g vs. 22.66 ± 1.35 g/100 g in EtAc vs. 18.72 ± 0.08 g/100 g in AcO and 15.50 ± 0.44 g/100 g in EtOH; *p* < 0.05). The variation of glycolipids in bagasse was inversely proportional: 60.15 ± 0.02 g/100 g in EtOH; 45.29 ± 0.34 g/100 g in AcO; 35.28 ± 1.07 g/100 g in EtAc; and 9.76 ± 0.28 g/100 g in DCM (*p* < 0.05). 

Glycolipids are interesting compounds that are mainly microbiological in origin but are also produced by photosynthetic organisms [38]. They have been successfully assayed to avoid *Listeria monocytogenes*’ biofilms in milk and cheese [39]. Additionally, rhamnolipids are biosurfactants with remodelling lipid properties in plasma through interaction with lipid rafts [40] and in lipid–protein complexes involved in cell signalling as well as in transmembrane transport. Since research on glycolipids may bring new health and technological applications, finding new sources and ways to obtain enriched extracts may help in future studies.

As commented above, Asikin et al. [15] reported that aldehydes and sterol esters were the main lipids in sugarcane rinds followed by TGs, sterols and FFAs. According to the obtained results, FFA was the third most present group of lipids in terms of concentration. EtAc and DCM samples had similar values (19.60 ± 1.53 g FFA/100 g and 18.63 ± 2.17 g FFA/100 g, respectively), as it was also observed for AcO (15.04 ± 1.35 g FFA/100 g) and EtOH (14.58 ± 0.73 g FFA/100 g). For bagasse extracts, FFA amounts were lower than in straw samples. Thus, DCM (13.99 ± 1.08 g/100 g) and EtAc (12.60 ± 0.37 g/100 g) were the more efficient solvents to recover such compounds. EtOH samples had contents of 5.51 ± 0.34 g/100 g while AcO exerted a significantly better performance (10.05 ± 0.13 g/100 g). 

Regarding TG, the contents were much lower than those registered for FFA. As expected, due to their immiscibility with water, it was revealed that DCM (5.12 ± 0.27 g/100 g straw lipids; 2.55 ± 0.16 g/100 g bagasse lipids) and EtAc (4.48 ± 0.26 g/100 g straw lipids; 2.25 ± 0.11 g/100 g bagasse lipids) are the best solvents to recover TG for both straw and bagasse. However, AcO (4.08 ± 0.04 g/100 g straw lipids; 2.24 ± 0.08 g/100 g bagasse lipids) showed contents close to those found when using EtAc, although differences were only significant in the case of sugarcane straw. Previous studies of wild-type sugarcane showed TG contents of 4.76% [27], therefore agreeing with the concentrations found here. 

In the current reported results, the presence of other acylglycerides such as monoglycerides and DGs was detected which was not detected in the works of both Asikin et al. [15] and Huang et al. [27]. 

Obtained data showed the presence of PLSs (i.e., PI, PS and PC) in both sugarcane straw and bagasse but only when assaying EtOH and AcO (extracts of the latter solvent in bagasse samples did not show the presence of PLSs). The total PLS content in EtOH was 1.54 ± 0.01 g/100 g straw lipids and 0.69 ± 0.08 g/100 g bagasse lipids while it was 0.43 ± 0.01 g/100 g straw lipids in AcO. Different methods of isolating phospholipids have been discussed elsewhere, comparing those assaying EtOH, methanol, AcO or even acetonitrile, concluding that AcO is an excellent solvent to recover PLSs as they are precipitated [34]. However, those kinds of methodusually use chilled AcO, explaining why, in the current results, phospholipids are present in such extracts although in lower concentrations than when using EtOH [37]. Moreover, those works describing PLS recovery by crystallisation also purified the extract dissolving these compounds in EtOH [37]. 

In the current study, in straw samples, the PLS fraction was represented by PI, PS and PC but only this latter compound was detected in bagasse and only when assaying EtOH. In our results, PC was the main PLS in the straw samples. However, other authors found a different distribution as PI was the main compound and PC and PE were in similar concentrations [27]. It must be noted that samples in the assayed study were obtained from cultivars in Brazil while the aforementioned bibliographic data were from samples obtained in the USA. Differences in the cultivars, crop season and geographical situation can affect the composition of the plant.

FOHs are an interesting group of compounds that can be found in different plant materials such as rice bran [41] and other plant tissues [42], among which sugarcane is an interesting source [13,15,20]. Such compounds have recently attracted increasing attention due their promising effect to restore sleep alterations by stress in mice [18] or their capacity to improve the intimal lesions of the aorta, suggesting anti-inflammatory potential [43].

When comparing the different straw and bagasse extracts, it was observed that the fatty alcohols concentrations for EtOH samples were 7.14 ± 0.12 g/100 g in straw and 8.56 ± 0.01 g/100 g in bagasse. Furthermore, in the samples obtained after assaying AcO, the fatty alcohols concentrations were 11.32 ± 0.55 g/100 g for straw and 11.78 ± 0.02 g/100 g for bagasse. However, when using EtAc, the fatty alcohols content was 8.24 ± 0.05 g/100 g for straw and 14.17 ± 0.22 g/100 g. In general, bagasse had higher fatty alcohols content than straw.

According to the obtained results, in the lipid profile, DCM showed the best performance results for both sugarcane straw and bagasse in terms of the contents of hydrocarbons, FFAs, FOHs, esters, phytosterols and acylglycerols (i.e., TGs, MGs and DGs). It was also found that the lipid proportion when assaying AcO, EtAc and EtOH for some compounds (i.e., DG) was affected by the matrix. 

Finally, glycolipids and PLSs where mainly found in EtOH, therefore suggesting that, for this kind of samples, this solvent is the best option. 

Lipid isolation is a complex task and indeed, several different methods based in chloroform/methanol, isopropanol/hexane and methyl tert-butyl ether/methanol have been proposed and are widely used to extract such compounds [34], such as recently proposed single-layer methods showing a similar performance [44]. However, the selection of the procedure and solvent system is a crucial step as these can affect the qualitative and quantitative composition of the extract [28], and in the case of dairy products, it has been demonstrated that such choices can specifically impact the phospholipids fraction [45]. Thus, the observed variations in the isolation capacity of the solvents tested in the current work are consistent with the findings of the existing scientific literature. 

Moreover, studies to describe how the distribution of lipids is affected by solvents can be useful to understand the interaction with the sample and select the most suitable procedure accordingly, specifically if a group of compounds is of interest.

### 3.3. Extract Characterisation by GC–MS 

The results commented upon and discussed in the last section regarding the lipid classes composition of the EtOH, AcO, EtAc, DCM extracts show how each solvent affects the different groups of compounds. However, we were interested in obtaining information about individual compounds (i.e., FFAs, hydrocarbons and FOHs) and thus a GC–MS analysis was conducted. For both sugarcane straw (Table 3) and bagasse (Table 4) samples, the same groups were detected such as FFAs, hydrocarbons, FOHs, and STs. Phenolic compounds were also detected (i.e., coumaric acid), aldehydes (FALs) as octacosanal (FAL 18:0) and terpenes (only in straw samples; friedelan-3-one). During the analyses, other compounds were detected as polyols, in addition to d-erythrotetrofuranose, levoglucosan and some other sugars that were not possible to identify (Supplementary Material Table S2). Moreover, the obtained data show that, regarding polyols, DCM was only able to isolate glycerol and 1,2,3-butanetriol.

The obtained data from the HPLC–ELSD analyses showed that DCM had a good capability for isolating non-polar lipids while EtOH was more suitable for recovering polar compounds such as phospholipids and glycolipids. While these analyses give information about the distribution of the different lipid subfamilies, through GC–MS, it is possible to gather individualised data, at least from those compounds that can be volatilised. 

Thus, as expected, DCM had the highest contents of FFAs in the straw extracts as the contents were of 66.73 ± 9.20 g/kg while they were 52.38 ± 2.84 g/kg, 38.24 ± 3.65 g/kg and 15.26 g/kg, respectively (*p* < 0.05), for EtAc, AcO and EtOH. On the other hand, for bagasse samples, the amounts were as follows: 35.38 ± 2.94 g/kg AcO extract ≈ 31.92 ± 3.14 g/kg EtAc extract ≈ 26.36 ± 3.22 g/kg DCM extract > 9.98 ± 1.68 g/kg EtOH extract (*p* < 0.05). However, it must be noted that, for bagasse, the main FFA was octacosanoic acid (FFA 28:0), a long-chain saturated fatty acid. Samples collected when using AcO and DCM, FFA 28:0 concentrations were not significantly different (13.82 ± 1.23 g/kg vs. 13.81 ± 1.50 g/kg, respectively) but were lower for EtAc (11.09 ± 2.61 g/kg). The solvent affected the quantitative profile of the samples in the case of EtOH as the main FFA was palmitic acid (FFA 16:0; 2.86 ± 0.62 g/kg bagasse extract) instead of octacosanoic (2.84 ± 0.90 g/kg bagasse extract). Such results suggest, as observed in the lipid classes’ analyses, that the matrix can affect the isolation of the compounds.

Previously available information reported that the total FFA concentration when lipids were isolated through supercritical fluids (SFE) was 1.5 g/kg straw and 0.3 g/kg bagasse [13], while those research works assaying isolation with acetone found contents of 1.2 g/kg straw and 0.1 g/kg bagasse [20]. Although those values are lower than those found in the present study, several factors can affect the lipid composition: from those exclusively related to the crop (season, cultivar, management system) to those associated with isolation and analysis (i.e., conditions, equipment). Thus, FFA distribution in the elsewhere obtained acetone extract was mainly composed of palmitic acid (FFA 16:0) while linolenic (FFA 18:2 c9c12) and FFA 28:0 were present in similar concentrations [20]. However, FFA 28:0 was predominantly present in bagasse lipids. On the other hand, in the investigations of Attard et al. [13], FFA C28 was in trace amounts in straw samples while bagasse showed very low contents (i.e., 0.06 g/kg).

In the fraction of FOHs, 1-dotriacontanol (FOH 32:0) was the featured compound in straw isolates varying from 19.43 ± 2.56 g/kg DCM extract to 1.39 ± 0.28 g/kg EtOH extract. In bagasse, this moiety was characterised by 1-octacosanol and the values ranged from 21.64 ± 2.07 g/kg DCM extract to 5.89 ± 1.16 g/kg EtOH extract. 

The FAL equivalent of this compound (i.e., octacosanal, FAL C28:0) was found in quantities of 9.22 ± 1.38 g/kg DCM straw extract and 13.79 ±1.65 g/kg DCM bagasse extract. The rest of the solvents accounted for significantly lower contents in both straw and bagasse lipids. This distribution agrees with that previously reported by Attard et al. [13], although the concentrations (0.3 g FOH 32:0/kg straw and 0.7 g FOH 18:0/kg bagasse) observed by those authors were lower than those reported herein. Furthermore, data from Del Rio et al. [20] concluded that FOH 28:0 was the main FOH in both straw and bagasse.

The already commented research works as well as the composition presented here found a similar FOH profile comprising from FOH C14:0 to FOH C34:0. However, other authors described in sugarcane rind from different cultivars, a profile comprising compounds from FOH 22:0 to FOH 30:0 where FOH 18:0 was the featured compound. In those works, contents showed high variability when comparing cultivars (i.e., from 1 g/kg to 0.5 g/kg) [15].

Thus, the differences in the quantities may be related to the isolation method. For example, in straw, the utilisation of EtOH and EtAc showed contents 2-fold higher than those reported in the bibliography and such variation could be further associated with differences among the used cultivar of this present work and those already reported. 

Both FFAs and FOHs were mainly composed of long saturated compounds. This aspect was also observed to be continued in the group of hydrocarbons. Straw DCM extracts were composed of pentacosane (C25:0; 0.40 ± 0.08 g/kg), heptacosane (C27:0; 0.94 ± 0.06 g/kg), nonacosane (C29:0; 1.32 ± 0.20 g/kg) and hentriacontane (C31:0; 2.18 ± 0.58 g/kg). Nevertheless, pentacosane was not detected in the other extracts. For AcO and EtAc, the contents for the rest of hydrocarbons were significantly lower than when testing DCM. In EtOH extracts, hentriacontane was the only hydrocarbon detected (0.28 ± 0.05 g/kg). 

According to the data obtained from bagasse samples, when comparing the results among the different solvents, it was clear that DCM extracts were also enriched in hydrocarbons, mainly in nonacosane (2.87 ± 2.22 g/kg DCM extract vs. 0.53 ± 0.13 g/kg AcO extract vs. 0.35 ± 0.09 g/kg EtAc extract). This compound was not detected in the EtOH samples.

To the best of our knowledge, from the works reporting the composition in lipophilic phytochemicals from sugarcane materials, only the works assaying SFE reported the composition in hydrocarbons [13]. Those results suggested that straw lipids were characterised by the presence of tritriacontane (C33:0; 0.3 g/kg), while for bagasse, was C31:0 (0.03 g/kg). As commented above with other fractions, variations can be explained on the basis of the isolation procedures and cultivars.

As the assayed by-products in the present research work were plant materials, the presence of phytosterols was expected. In samples obtained from straw, total contents were 34.16 ± 2.73 g/kg in the DCM extract, 11.07 ± 1.73 g/kg in the AcO extract, 9.55 ± 0.61 g/kg EtAc extract and 4.06 ± 0.63 g/kg in the EtOH extract. This fraction was composed of campesterol (ST C28:1;O), stigmasterol (ST 29:2;O), β-sitosterol (ST 29:1;O) and stigmast-4-en-3-one (ST29:2;O2). Regarding bagasse, there were only slight differences in the total sterol’s concentrations between DCM (28.22 ± 1.17 g/kg), AcO (26.33 ± 2.69 g/kg) and EtAc (24.59 ± 3.10 g/kg). 

From previous research works reporting ST composition, it can be concluded that there is a high variability in the contents and distribution of this group of compounds in the lipids of sugarcane straw and bagasse. Thus, in acetone isolates, this fraction was characterised by sitosterol (0.1 g/kg straw lipids; 0.01 g/kg bagasse lipids) [20]. Furthermore, the utilisation of SFE led to phytosterol distributions where, for straw lipids, β-sitosterol was in levels of 0.6 g/kg and stigmasterol in 0.5 g/kg, while for bagasse, the amounts were 0.1 g/kg and 0.08 g/kg, respectively [13]. Interestingly, such contents testing SFE to recover straw lipids were close to the results obtained in this investigation assaying EtOH. On the other hand, as a supercritical fluid, only CO_2_ was used without EtOH as modifier or co-solvent.

Finally, the capacity of EtOH to isolate polar compounds was highlighted by the presence of coumaric acid (1.64 g/kg straw extract; 36.11 g/kg bagasse extract) as these samples contained significantly higher levels than those obtained through AcO or EtAc. This compound was not detected in DCM.

### 3.4. FTIR-ATR Results

**Figure 3:**
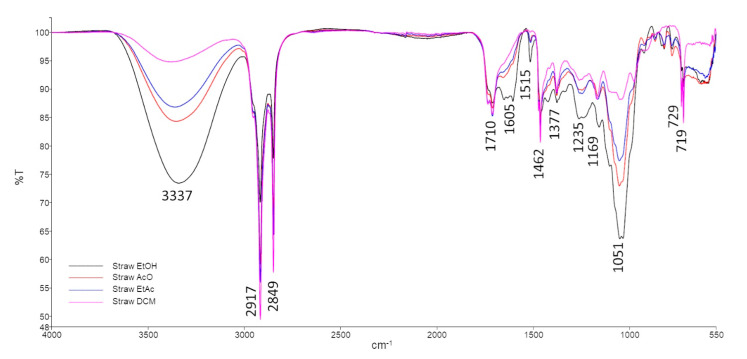
FTIR spectra of straw extracts.

**Figure 4:**
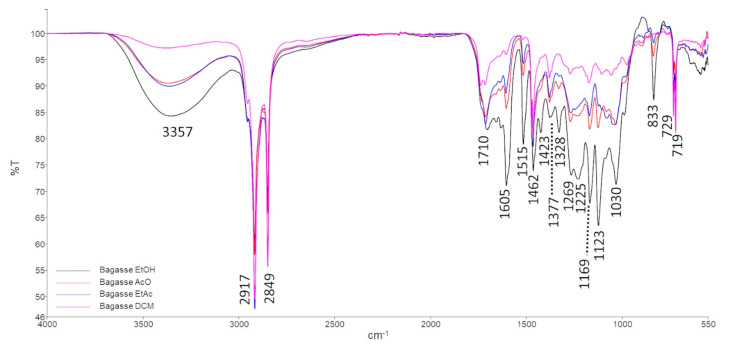
FTIR spectra of bagasse extracts.

The FTIR-ATR overlapping spectra of straw (Figure 3) and bagasse (Figure 4) extracts from different solvents (EtOH, AcO, EtAc and DCM) revealed that the composition of the obtained waxes was quite similar. The vibrational bands were identified based on literature [46] and are resumed in Table 5.

The vibrational bands at 3357–3337 cm^−1^, 1710 cm^−1^, 1169 cm^−1^ and 1051 cm^−1^ are related to the -OH stretching and bending vibrations and C-O asymmetric and symmetric stretching vibrations, respectively. These vibrations can be produced by alcohol groups, which agrees with the HPLC–ELSD and GC–MS results that identified several FOHs and phytosterols.

Moreover, FTIR-ATR spectra also showed three vibrational bands related to amine groups, at 1605 cm^−1^, 1269–1225 cm^−1^ and 1123 cm^−1^ (corresponding to the RONH_2_ functional group, NH_2_ rocking/twisting and N-H bending vibrations, respectively) and a band at 1515 cm^−1^ from the C-N stretching vibration of the amides functional group. FTIR-ATR vibrational bands associated with amines and amides were probably due to the presence of phospholipids such as PI and PS (amine functional group) and glycolipids.

Additionally, a band at 1328 cm^−1^, associated with the –CH deformation vibration on a –CHO functional group (aldehyde), present in the bagasse extracts, is also in accordance with the compositional identification obtained by HPLC–ELSD and GC–MS, which reveal the presence of octacosanal. Although this compound is also identified in straw waxes, in the FTIR-ATR spectra, it is not possible to identify these characteristic vibrational bands. This fact is probably due to the amount of octacosanal being much lower in straw extracts.

### 3.5. Differential Scanning Calorimetry (DSC) 

All straw and bagasse extracts, from the different solvents were solid at room temperature (waxes). The DSC analysis of these waxes allowed to determine its melting and crystallisation points, in addition to its oxidation and decomposition temperatures. The first heating cycle allowed to eliminate the samples’ thermal history [13]. Only the transitions observed during the cooling and the second heating cycles were considered. The results are summarised in Table 6. The enthalpies are presented in absolute values.

The crystallisation points of both straw and bagasse waxes are quite similar to one another. Thus, in the isolates from straw, the temperatures ranged from 65.9 °C in EtAc to 56.1 °C in AcO. On the other hand, regarding the crystallisation temperature in the bagasse extracts, the highest values were observed for DCM (62.9 °C) and EtAc (62.1 °C). Interestingly, the results for EtOH bagasse extract (57.9 °C) are similar to those recorded for straw isolates using this same solvent (57.8 °C).

The melting points varied from 66.4 °C (AcO straw extract) to 74.8 °C (EtAc straw extract) while in the bagasse samples, these ranged from 68.1 °C (EtOH) to 72.2 °C (DCM). According to the results obtained from the GC–MS analyses, it would be expected that, in the straw samples, the highest melting points were recorded for DCM extracts as the contents in fatty alkyls, FOHs and STs showed the highest levels in these samples. However, the results also show that glycerol and other polyols were poorly isolated by this solvent. Such compounds may have affected the obtained values. However, the enthalpies for the melting process in DCM was 55.1 J/g and 55.3 J/g for EtAc. 

When sugarcane wax from peel was obtained through Soxhlet with carbon tetrachloride, the melting temperature was registered at 62 °C [19]. Moreover, in waxes from sugarcane rind, leaves and bagasse, isolated through SFE, the melting points were 73 °C, 63 °C and 71 °C, respectively [13]. 

Regarding the values obtained for the melting points, the current lipid extracts can be considered waxes. Furthermore, for each wax, the enthalpies involved in the respective melting and crystallisation processes were also similar, suggesting that the crystallised fraction is completely melted when re-heated.

Still during the second heating cycle, an exothermic process occurred, at 163.6 °C or 168.0 °C, only for the waxes extracted with DCM (straw and bagasse extracts, respectively). Considering the composition of these waxes, and comparatively to the others, it was possible to verify that the waxes extracted with DCM do not present 4-coumaric acid in their composition. Since this compound has antioxidant properties, the fact that it is not present in DCM waxes facilitates its oxidation. Therefore, this exothermic peak may be associated with wax oxidation. 

When all waxes were heated above 350 °C, they decomposed. The lowest decomposition temperature was recorded for the straw ethanol wax (354.5 °C), while the highest was verified for the bagasse dichloromethane wax (436.4 °C). Rueda-Ordoñez et al. [47] also found a decomposition temperature of 350 °C, although those works were conducted in whole sugarcane straw.

The DSC curves showed very wide overlapping irregular peaks for decomposition, that are highly related to the complex composition of the waxes. This resulted into variable enthalpy values (65.7–279.5 J/g).

## 4. Conclusions

The obtained results confirm that each tested solvent interacted differently with sugarcane straw and bagasse matrices and the lipid composition of the extracts depended on the assayed extractant. 

Thus, although EtOH resulted in the highest extraction yield, the analyses also showed that other non-lipid compounds as polyols may be present in the isolates. Additionally, the data evidenced that the matrix influenced the lipids recovery since for straw samples, the solvents AcO, EtAc and DCM showed a decreasing isolating capacity, whilst in bagasse samples, those solvents led to similar yields.

Regarding the composition of the extracts, DCM was mostly efficient in recovering non-polar lipids. Moreover, AcO and EtAc showed similar capacities for non-polar lipids, however, when compared, only AcO recovered PLSs. Glycolipids were mainly found in the extracts obtained with EtOH, therefore suggesting its suitability for enriching extracts from sugarcane straw and bagasse in polar lipids. 

All isolates had long-chain alkyls and phytosterols in their composition but those produced by DCM had the highest concentrations in agreement with the lipophilic nature of this solvent. Thus, DCM was suitable for recovering the bioactive octacosanol (FOH 28:0) and phytosterols. Accordingly, in further investigations focused on unravelling the biological effects of these compounds from sugarcane biomass, DCM should be the solvent of choice. However, such obtained results must be carefully interpreted as this kind of reagent can interfere with lipid bioactivity and further product development may face restrictions by regulations. Finally, the presence of long chain alkyls and other high molecular weight lipids (i.e., TG and ST) results in melting points above 60 °C and samples are consistent with being waxes. 

In summary, the results of this research work enable us to conclude that sugarcane waxes from biomass such as straw and bagasse are complex lipid mixtures of polar and non-polar compounds. The extractant greatly influences the distribution of these compounds in the obtained sample. DCM will enrich the isolates in glycerolipids, FFAs, FOHs, FALs, STs and hydrocarbons. On the other hand, EtOH will yield isolates rich in glycolipids. Therefore, depending on the application and objectives of future research studies, the solvent to recover such lipids needs to be carefully selected.

## Figures and Tables

**Table 1:** Lipid classes composition (g/100 g of lipids) of different sugarcane straw extracts.

	STRAW							
Compound	EtOH	AcO	EtAc			DCM		
**Hydrocarbon**	24.36 ^d^ ± 0.06	31.95 ^ab^ ± 3.62	29.96 ^c^ ± 1.35			37.24 ^a^ ± 1.07		
**Wax esters**	2.60 ^d^ ± 0.04	3.70 ^b^ ± 0.06	2.84 ^c^ ± 0.01			4.24 ^a^ ± 0.01		
**Triglycerides**	3.66 ^d^ ± 0.04	4.08 ^c^ ± 0.04	4.48 ^b^ ± 0.26			5.12 ^a^ ± 0.27		
**Fatty alcohols**	7.14 ^c^ ± 0.12	11.32 ^a^ ± 0.55	8.24 ^b^ ± 0.05			11.50 ^a^ ± 0.10		
**Phytosterol**	3.58 ^ab^ ± 0.47	3.58 ^b^ ± 0.09	3.54 ^b^ ± 0.04			4.15 ^a^ ± 0.14		
**Diglycerides**	6.18 ^a^ ± 0.60	4.88 ^b^ ± 0.16	6.44 ^a^ ± 0.12			6.38 ^a^ ± 0.29		
**Free fatty acids**	14.58 ^c^ ± 0.73	15.04 ^bc^ ± 1.35	19.60 ^a^ ± 1.53			18.63 ^ab^ ± 2.17		
**Monoglycerides**	1.38 ^b^ ± 0.09	1.40 ^b^ ± 0.11	1.67 ^ab^ ± 0.09			2.18 ^a^ ± 0.22		
**Glycolipids**	34.98 ^a^ ± 1.66	23.62 ^b^ ± 2.18	23.25 ^b^ ± 1.00			10.55 ^c^ ± 1.83		
**Phosphatidylinositol**	0.44 ^a^ ± 0.04	0.17 ^b^ ± 0.02	n.d ^c^		n.a	n.d ^c^		n.a
**Phosphatidylserine**	0.45 ^a^ ± 0.09	0.20 ^b^ ± 0.07	n.d ^c^		n.a	n.d ^c^		n.a
**Phosphatidylcholine**	0.65 ^a^ ± 0.15	0.06 ^b^ ± 0.01	n.d ^c^		n.a	n.d ^c^		n.a

Results expressed as mean ± SD (*n* = 3). EtOH: ethanol; AcO: acetone; EtAc: ethyl acetate; DCM: dichloromethane. n.d: not detected; n.a: not applied. Different superscript letters in a row indicate statistically significant differences between solvents (*p* < 0.05).

**Table 2:** Lipid classes composition (g/100 g of lipids) of different sugarcane bagasse extracts.

	BAGASSE									
Compound	EtOH	AcO			EtAc			DCM		
**Hydrocarbon**	15.50 ^d^ ± 0.44	18.72 ^c^ ± 0.08			22.66 ^b^ ± 1.35			38.93 ^a^ ± 4.93		
**Wax esters**	1.48 ^d^ ± 0.08	1.80 ^c^ ± 0.05			2.08 ^b^ ± 0.22			5.28 ^a^ ± 0.07		
**Triglycerides**	1.73 ^c^ ± 0.02	2.24 ^c^ ± 0.08			2.25 ^b^ ± 0.11			2.55 ^a^ ± 0.16		
**Fatty alcohols**	8.56 ^d^ ± 0.01	11.78 ^c^ ± 0.02			14.17 ^b^ ± 0.22			19.80 ^a^ ± 0.91		
**Phytosterol**	1.96 ^c^ ± 0.07	2.77 ^b^ ± 0.01			3.61 ^a^ ± 0.19			3.70 ^a^ ± 0.13		
**Diglycerides**	3.19 ^b^ ± 0.08	4.48 ^a^ ± 0.05			4.67 ^a^ ± 0.06			4.18 ^a^ ± 0.68		
**Free fatty acids**	5.51 ^c^ ± 0.34	10.05 ^b^ ± 0.13			12.60 ^a^ ± 0.37			13.99 ^a^ ± 1.08		
**Monoglycerides**	1.22 ^b^ ± 0.21	2.87 ^a^ ± 0.20			2.68 ^b^ ± 0.01			1.80 ^b^ ± 0.09		
**Glycolipids**	60.15 ^a^ ± 0.02	45.29 ^b^ ± 0.34			35.28 ^c^ ± 1.07			9.76 ^d^ ± 0.28		
**Phosphatidylcholine**	0.69 ^a^ ± 0.08	n.d ^b^		n.a	n.d ^b^		n.a	n.d ^b^		n.a

Results expressed as mean ± SD (*n* = 3). EtOH: ethanol; AcO: acetone; EtAc: ethyl acetate; DCM: dichloromethane. n.d: not detected; n.a: not applied. Different superscript letters in a row indicate statistically significant differences between solvents (*p* < 0.05).

**Table 3:** Profile (g/kg) in free fatty acids, hydrocarbons, fatty alcohols, sterols, fatty aldehydes, and terpenes of different sugarcane straw extracts.

	STRAW											
	EtOH			AcO			EtAc			DCM		
**Octanoic acid (FFA 8:0)**	4.36 ^c^ ± 0.31			7.20 ^b^ ± 0.20			20.15 ^a^ ± 0.24			8.45 ^b^ ± 0.63		
**Dodecanoic acid (FFA 12:0)**	0.24 ^c^ ± 0.03			0.40 ^b^ ± 0.09			0.48 ^b^ ± 0.03			1.40 ^a^ ± 0.24		
**Myristic acid (FFA 14:0)**	0.49 ^c^ ± 0.10			1.20 ^a^ ± 0.12			0.64 ^b^ ± 0.07			1.25 ^a^ ± 0.21		
**Palmitic acid (FFA 16:0)**	4.89 ^c^ ± 0.49			13.33 ^b^ ± 1.08			12.58 ^b^ ± 0.43			19.15 ^a^ ± 0.32		
**Linoleic acid (FFA 18:1 c9c12)**	0.55 ^c^ ± 0.11			1.98 ^b^ ± 0.12			2.23 ^b^ ± 0.11			4.09 ^a^ ± 0.78		
**Oleic acid (FFA 18:1 c9)**	2.50 ^d^ ± 0.50			6.00 ^c^ ± 1.04			8.88 ^b^ ± 0.32			15.21 ^a^ ± 2.78		
**Stearic acid (FFA 18:0)**	1.69 ^c^ ± 0.22			4.01 ^b^ ± 0.55			4.12 ^b^ ± 0.17			6.58 ^a^ ± 1.20		
**Arachidic acid (FFA 20:0)**	0.31 ^c^ ± 0.05			1.50 ^ab^ ± 0.22			1.48 ^b^ ± 0.09			1.91 ^a^ ± 0.23		
**Behenic acid (FFA 22:0)**	n.d ^c^		n.a	0.40 ^b^ ± 0.14			0.46 ^b^ ± 0.02			0.69 ^a^ ± 0.14		
**Lignoceric acid (FFA 24:0)**	n.d ^c^		n.a	0.58 ^b^ ± 0.19			0.59 ^b^ ± 0.02			1.11 ^a^ ± 0.21		
**Octacosanoic acid (FFA 28:0)**	0.24 ^d^ ± 0.08			1.63 ^b^ ± 0.22			0.77 ^c^ ± 0.13			6.89 ^a^ ± 1.04		
**Σ FFA**	15.26 ^d^ ± 1.25			38.24 ^c^ ± 3.65			52.38 ^b^ ± 2.84			66.73 ^a^ ± 9.20		
**1-Octacosanol (FOH 28:0)**	2.35 ^c^ ± 0.35			5.58 ^b^ ± 0.16			2.28 ^c^ ± 0.09			18.19 ^a^ ± 2.52		
**1-Triacontanol (FOH 30:0)**	1.12 ^b^ ± 0.22			3.52 ^c^ ± 0.15			1.65 ^b^ ± 0.11			13.84 ^a^ ± 1.46		
**1-Dotriacontanol (FOH 32:0)**	1.39 ^b^ ± 0.28			4.48 ^c^ ± 0.21			2.01 ^b^ ± 0.22			19.43 ^a^ ± 2.56		
**1-Tetratriacontanol (FOH 34:0)**	n.d ^c^		n.a	0.35 ^b^ ± 0.06			n.d ^c^		n.a	2.17 ^a^ ± 0.22		
**Σ FOH**	4.86 ^c^ ± 0.89			13.93 ^b^ ± 1.14			5.94 ^c^ ± 0.37			53.63 ^a^ ± 6.73		
**Pentacosane (C25:0)**	n.d ^b^		n.a	n.d ^b^		n.a	n.d ^b^		n.a	0.40 ^a^ ± 0.08		
**Heptacosane (C27:0)**	n.d ^d^		n.a	0.45 ^b^ ± 0.08			0.28 ^c^ ± 0.01			0.94 ^a^ ± 0.06		
**Nonacosane (C29:0)**	n.d ^d^		n.a	0.47 ^b^ ± 0.07			0.24 ^c^ ± 0.02			1.32 ^a^ ± 0.20		
**Hentriacontane (C31:0)**	0.28 ^d^ ± 0.05			0.97 ^b^ ± 0.15			0.49 ^c^ ± 0.05			2.18 ^a^ ± 0.58		
**Σ HYDROCARBONS**	0.28 ^d^ ± 0.05			1.89 ^b^ ± 0.15			1.01 ^c^ ± 0.07			4.84 ^a^ ± 0.91		
**Campesterol (ST 28:1;O)**	0.43 ^c^ ± 0.06			0.89 ^b^ ± 0.19			0.94 ^b^ ± 0.08			2.87 ^a^ ± 0.57		
**Stigmasterol (ST 29:2;O)**	0.89 ^c^ ± 0.11			3.59 ^b^ ± 0.34			2.16 ^b^ ± 0.05			11.81 ^a^ ± 1.62		
**β-Sitosterol (ST 29:1;O)**	1.56 ^c^ ± 0.09			3.97 ^b^ ± 0.69			3.96 ^b^ ± 0.35			11.00 ^a^ ± 2.01		
**Stigmast-4-en-3-one (ST 29:2;O2)**	1.17 ^c^ ± 0.03			2.62 ^b^ ± 0.34			2.49 ^b^ ± 0.15			8.49 ^a^ ± 0.99		
**Σ ST**	4.06 ^c^ ± 0.63			11.07 ^b^ ± 1.73			9.55 ^b^ ± 0.61			34.16 ^a^ ± 2.73		
**4-Coumaric acid**	1.63 ^a^ ± 0.26			0.62 ^b^ ± 0.09			0.35 ^c^ ± 0.05			n.d ^d^		n.a
**Octacosanal (FAL 28:0)**	0.25 ^c^ ± 0.05			1.70 ^b^ ± 0.20			0.39 ^c^ ± 0.02			9.22 ^a^ ± 1.38		
**Friedelan-3-one**	0.61 ^c^ ± 0.08			1.52 ^b^ ± 0.32			1.39 ^b^ ± 0.11			4.65 ^a^ ± 0.91		

Results expressed as mean ± SD (*n* = 3). EtOH: ethanol; AcO: acetone; EtAc: ethyl acetate; DCM: dichloromethane. FFAs: free fatty acids; FOHs: fatty alcohols; STs: sterols; FALs: fatty aldehydes; n.d: not detected; n.a: not applied. Different superscript letters in a row indicate statistically significant differences between solvents (*p* < 0.05).

**Table 4:** Profile (g/kg) in free fatty acids, hydrocarbons, fatty alcohols, sterols, fatty aldehydes and terpenes of different sugarcane bagasse extracts.

	BAGASSE											
	EtOH			AcO			EtAc			DCM		
**Dodecanoic acid (FFA 12:0)**	n.d ^c^		n.a	0.16 ^b^ ± 0.02			0.37 ^a^ ± 0.08			n.d ^c^		n.a
**Myristic acid (FFA 14:0)**	n.d ^b^		n.a	0.40 ^a^ ± 0.02			n.d ^b^		n.a	n.d ^b^		n.a
**Palmitic Acid (FFA 16:0)**	2.86 ^c^ ± 0.62			6.52 ^a^ ± 0.86			6.92 ^a^ ± 0.62			4.33 ^b^ ± 0.58		
**Stearic acid (FFA 18:0)**	1.03 ^b^ ± 0.16			3.68 ^a^ ± 0.62			2.70 ^a^ ± 0.23			1.77 ^b^ ± 0.17		
**Oleic Acid (FFA 18:1 c9)**	1.57 ^c^ ± 0.47			4.50 ^a^ ± 0.06			4.37 ^a^ ± 0.17			2.49 ^b^ ± 0.28		
**Linoleic acid (FFA 18:2 c9c12)**	0.87 ^b^ ± 0.32			2.49 ^a^ ± 0.35			2.47 ^a^ ± 0.09			1.13 ^b^ ± 0.25		
**Arachidic acid (FFA 20:0)**	0.44 ^c^ ± 0.08			1.27 ^a^ ± 0.16			1.28 ^a^ ± 0.17			0.92 ^b^ ± 0.19		
**Behenic acid (FFA 22:0)**	n.d ^c^		n.a	0.88 ^a^ ± 0.13			0.96 ^a^ ± 0.14			0.48 ^b^ ± 0.11		
**Lignoceric acid (FFA 24:0)**	0.38 ^c^ ± 0.03			1.66 ^a^ ± 0.18			1.76 ^a^ ± 0.36			1.43 ^a^ ± 0.24		
**Octacosanoic acid (FFA 28:0)**	2.84 ^c^ ± 0.90			13.82 ^a^ ± 1.23			11.09 ^b^ ± 2.61			13.81 ^ab^ ± 1.50		
**Σ FFA**	9.98 ^c^ ± 1.68			35.38 ^a^ ± 2.94			31.92 ^ab^ ± 3.14			26.36 ^b^ ± 3.22		
**1-Octacosanol (FOH 28:0)**	5.89 ^c^ ± 1.16			19.64 ^a^ ± 1.92			11.21 ^b^ ± 2.81			21.64 ^a^ ± 2.07		
**1-Triacontanol (FOH 30:0)**	1.29 ^d^ ± 0.21			3.67 ^b^ ± 0.59			2.21 ^c^ ± 0.51			4.31 ^a^ ± 0.22		
**1-Dotriacontanol (FOH 32:0)**	0.68 ^d^ ± 0.15			2.01 ^b^ ± 0.44			1.16 ^c^ ± 0.27			3.21 ^a^ ± 0.21		
**Σ FOH**	7.85 ^c^ ± 1.54			25.32 ^a^ ± 2.74			14.59 ^b^ ± 3.33			29.16 ^a^ ± 2.40		
**Heptacosane (C27:0)**	0.36 ^c^ ± 0.10			1.67 ^a^ ± 0.17			0.99 ^b^ ± 0.21			2.15 ^a^ ± 0.36		
**Octacosane (C28:0)**	n.d ^b^		n.a	n.d ^b^		n.a	0.41 ^a^ ± 0.07			0.20 ^a^ ± 0.04		
**Nonacosane (C29:0)**	n.d ^d^		n.a	0.53 ^b^ ± 0.13			0.35 ^c^ ± 0.09			2.87 ^a^ ± 2.22		
**Hentriacontane (C31:0)**	n.d ^b^		n.a	0.46 ^a^ ± 0.03			n.d ^b^		n.a	n.d ^b^		n.a
**Σ HYDROCARBONS**	0.36 ^d^ ± 0.10			2.66 ^b^ ± 0.08			1.75 ^c^ ± 0.30			5.23 ^a^ ± 0.44		
**Campesterol (ST 28:1;O)**	0.65 ^c^ ± 0.08			1.87 ^a^ ± 0.16			1.78 ^a^ ± 0.15			1.33 ^b^ ± 0.19		
**Stigmasterol (ST 29:2;O)**	1.48 ^c^ ± 0.36			5.77 ^b^ ± 0.75			4.55 ^b^ ± 0.88			7.06 ^a^ ± 0.75		
**β-Sitosterol (ST 29:1;O)**	1.83 ^c^ ± 0.29			5.82 ^a^ ± 0.70			5.87 ^a^ ± 0.55			4.31 ^b^ ± 0.37		
**4,22-Stigmastadiene-3-one (ST 29:2;O)**	0.86 ^b^ ± 0.15			3.34 ^a^ ± 0.35			3.11 ^a^ ± 0.43			3.94 ^a^ ± 0.58		
**Stigmast-4-en-3-one (ST 29:2;O2)**	2.68 ^c^ ± 0.48			9.53 ^b^ ± 0.98			9.27 ^b^ ± 1.18			11.59 ^a^ ± 1.72		
**Σ ST**	7.50 ^c^ ± 1.04			26.33 ^ab^ ± 2.69			24.59 ^b^ ± 3.10			28.22 ^a^ ± 1.17		
**4-Coumaric acid**	36.11 ^a^ ± 2.13			11.29 ^b^ ± 0.30			7.93 ^c^ ± 0.71			n.d ^d^		n.a
**Octacosanal (FAL 28:0)**	2.27 ^d^ ± 0.84			7.91 ^b^ ± 0.08			4.40 ^c^ ± 1.27			13.79 ^a^ ± 1.65		

Results expressed as mean ± SD (*n* = 3). EtOH: ethanol; AcO: acetone; EtAc: ethyl acetate; DCM: dichloromethane. FFAs: free fatty acids; FOHs: fatty alcohols; STs: sterols; FALs: fatty aldehydes; n.d: not detected; n.a: not applied. Different superscript letters in a row indicate statistically significant differences between solvents (*p* < 0.05).

**Table 5:** FTIR frequencies interpretation table.

Wavenumber (cm^−1^)	Origin	Assignment
3357–3337	-OH stretching	Alcohols
2917	C-H stretching (-CH_3_, -CH_2_ and -CH)	Aliphatic chains
2849		
1710	-OH bending	Alcohols
1605	RONH_2_	Amines
1515	C-N stretching	Amides
1462	C-H bending (-CH_3_, -CH_2_)	Aliphatic chains
1423		
1377		
1328	-CHO (CH deformation)	Aldehydes
1269–1225	NH_2_ rocking/twisting	Amines
1169	C-O asymmetric stretching	Alcohols
1123	N-H bending	Amines
1051–1030	C-O stretching	Alcohols
833	CH_2_ rocking	Aliphatic chains
729	Rotational deformation of CH_2_ in chain	High aliphatic chains
719		

**Table 6:** Crystallisation, fusion, oxidation and decomposition temperatures (°C) and enthalpies (J/g) of sugarcane straw and bagasse.

	**Temperature (°C) (|ΔH| (J/g))**			
**Straw Extract**	**Crystallisation**	**Melting**	**Oxidation**	**Decomposition**
**EtOH**	57.8 (17.1)	70.1 (20.4)	n/a	354.5 (186.2)
**AcO**	56.1 (22.4)	66.4 (26.2)	n/a	410.6 (65.7)
**EtAc**	65.9 (53.7)	74.8 (55.3)	n/a	374.4 (153.6)
DCM	58.7 (45.2)	70.8 (55.1)	163.6 (23.4)	393.5 (225.6)
	**Temperature (°C) (|ΔH| (J/g))**			
**Bagasse Extract**	**Crystallisation**	**Melting**	**Oxidation**	**Decomposition**
**EtOH**	57.9 (11.6)	68.1 (12.4)	n/a	362.9 (279.5)
**AcO**	58.6 (43.5)	68.9 (51.1)	n/a	406.9 (176.9)
**EtAc**	62.1 (64.2)	68.6 (71.7)	n/a	393.2 (231.7)
**DCM**	62.9 (90.3)	72.2 (106.9)	168.0 (35.1)	436.4 (267.3)

n/a—not applicable.

## Data Availability

Not applicable.

## Supplementary Materials

The following supporting information can be downloaded at: https://www.mdpi.com/article/10.3390/foods11172661/s1, Table S1: HPLC–ELSD mobile phase gradient (%); Table S2: Non-lipidic compounds (g/kg) detected by GC–MS in the assayed sugarcane extracts.

## Abbreviations

Phospholipids (PLSs); triglycerides (TGs); diglycerides (DGs); free fatty acids (FFAs); fatty alcohols (FOHs); supercritical fluid extraction (SFE); octacosanol (FOH 28:0); dichloromethane (DCM); ethanol (EtOH); acetone (AcO); ethyl acetate (EtAc); triethylamine (TEA); N, O-Bis(trimethylsilyl) trifluoroacetamide with 1% trimethylchlorosilane (BSTFA); trimethylsilyl derivatives (TMSs); fatty aldehydes (FALs); sterols (STs); phosphatidylethanolamine (PE); phosphatidylcholine (PC); phosphatidylinositol (PI); tritriacontane (C33:0); campesterol (ST C28:1;O); stigmasterol (ST 29:2;O); β-sitosterol (ST 29:1;O); and stigmast-4-en-3-one (ST 29:2;O2).
