# Supplementary material for: Suitability of Solvent-Assisted Extraction for Recovery of Lipophilic Phytochemicals in Sugarcane Straw and Bagasse
Source: Foods. 2022 Sep 1;11(17):2661. doi: 10.3390/foods11172661 (PMC9455893; doi:10.3390/foods11172661)
Supplement: Supplementary file 1 [file foods-11-02661-s001.zip › foods-1865607-supplementary.pdf]

Table S1. HPLC-ELSD Mobile phase gradient (%)

Time (min.)	Mobile phase (percent)				Flow rate (mL/min.)
	A	B	C	D	
0	100	0	0	0	0.275
1.5	100	0	0	0	0.275
1.6	97	3	0	0	0.275
9	94	6	0	0	0.275
11	70	30	0	0	0.275
14	45	55	0	0	0.275
15	45	55	0	0	0.275
16	40	55	5	0	0.275
20	35	55	10	0	0.275
20.1	33	50	17	0	0.275
25	38	45	17	0	0.275
25.1	48	35	17	0	0.275
30	53	30	17	0	0.275
40	40	0	60	0	0.275
40.1	0	100	0	0	0.275
42	0	100	0	0	0.275
42.1	50	0	0	50	0.275
45	50	0	0	50	0.275
47	100	0	0	0	0.275
55	100	0	0	0	0.275

Table S2. Non-lipidic compounds (g/kg) detected by GC-MS in the assayed sugarcane extracts

	STRAW			
	EtOH	AcO	EtAc	DCM
**Unk Polyol**	n.d^{c} n.a	8.80^{a} ± 2.12	0.43^{b} ± 0.06	n.d^{c} n.a
**Glycerol**	20.59^{b} ± 1.93	46.48^{a} ± 0.37	48.86^{a} ± 1.77	27.14^{b} ± 4.51
**1,2,3-Butanetriol**	2.60^{c} ± 0.16	4.49^{b} ± 0.66	5.40^{a} ± 0.73	n.d^{d} n.a
**meso-Erythritol**	0.94^{c} ± 0.09	6.11^{a} ± 0.37	3.54^{b} ± 0.52	n.d^{d} n.a
**Ribitol**	n.d^{b} n.a	1.06^{a} ± 0.04	n.d^{b} n.a	n.d^{b} n.a
**Unk Sugar 1**	0.98^{bc} ± 0.08	1.13^{ab} ± 0.14	0.87^{c} ± 0.09	n.d^{d} n.a
**Unk Sugar 2**	n.d^{b} n.a	1.42^{a} ± 0.16	1.64^{a} ± 0.26	n.d^{b} n.a
**d-Erythrotetrofuranose**	1.01^{b} ± 0.17	1.34^{a} ± 0.04	0.41^{c} ± 0.09	n.d^{d} n.a
**Levoglucosan**	0.53^{b} ± 0.07	0.92^{a} ± 0.04	n.d^{c} n.a	n.d^{d} n.a
	**BAGASSE**			
	**EtOH**	**AcO**	**EtAc**	**DCM**
**Glycerol**	2.14^{b} ± 0.66	4.55^{a} ± 0.43	2.77^{b} ± 0.06	0.55^{c} ± 0.13
**1,2,3-Butanetriol**	8.29^{b} ± 1.39	6.43^{b} ± 0.18	15.27^{a} ± 1.25	0.41^{c} ± 0.07
**meso-Erythritol**	4.40^{c} ± 0.32	31.28^{a} ± 1.27	9.29^{b} ± 0.66	n.d^{d} n.a
**Erythropentitol**	n.d^{c} n.a	1.34^{a} ± 0.27	0.54^{b} ± 0.08	n.d^{c} n.a
**trans-Sinapyl alcohol**	1.83^{a} ± 0.17	n.d^{b} n.a	n.d^{b} n.a	n.d^{b} n.a
**Unk Sugar 3**	3.30^{a} ± 0.62	n.d^{b} n.a	2.95^{a} ± 0.12	n.d^{b} n.a
**Ribitol**	1.18^{b} ± 0.25	6.21^{a} ± 0.99	1.47^{b} ± 0.34	n.d^{d} n.a
**d-Erythrotetrofuranose**	n.d^{b} n.a	0.54^{a} ± 0.05	n.d^{b} n.a	n.d^{b} n.a

n.d: not detected; n.a: not applied; unk: unknown. Different superscript letters in a row indicate statistically significant differences between solvents ($p<0.05$).
